# HE4 and CA125 as a diagnostic test in ovarian cancer: prospective validation of the Risk of Ovarian Malignancy Algorithm

**DOI:** 10.1038/sj.bjc.6606092

**Published:** 2011-02-08

**Authors:** T Van Gorp, I Cadron, E Despierre, A Daemen, K Leunen, F Amant, D Timmerman, B De Moor, I Vergote

**Affiliations:** 1 Division of Gynaecological Oncology, Department of Obstetrics and Gynaecology, Universitaire Ziekenhuizen Leuven, Katholieke Universiteit Leuven, Herestraat 49, 3000 Leuven, Belgium; 2 Division of Gynaecological Oncology, Department of Obstetrics and Gynaecology, MUMC+, GROW – School for Oncology and Developmental Biology, PO Box 5800, 6202AZ Maastricht, The Netherlands; 3 Department of Electrical Engineering, ESAT-SCD/SISTA, Katholieke Universiteit Leuven, Kasteelpark Arenberg 10, 3001 Leuven-Heverlee, Belgium

**Keywords:** HE4, CA125, ovarian cancer, Risk of Ovarian Malignancy Algorithm

## Abstract

**Background:**

Recently, a Risk of Ovarian Malignancy Algorithm (ROMA) utilising human epididymis secretory protein 4 (HE4) and CA125 successfully classified patients as presenting a high or low risk for epithelial ovarian cancer (EOC). We validated this algorithm in an independent prospective study.

**Methods:**

Women with a pelvic mass, who were scheduled to have surgery, were enrolled in a prospective study. Preoperative serum levels of HE4 and CA125 were measured in 389 patients. The performance of each of the markers, as well as that of ROMA, was analysed.

**Results:**

When all malignant tumours were included, ROMA (receiver operator characteristic (ROC)-area under curve (AUC)=0.898) and HE4 (ROC-AUC)=0.857) did not perform significantly better than CA125 alone (ROC–AUC=0.877). Using a cutoff for ROMA of 12.5% for pre-menopausal patients, the test had a sensitivity of 67.5% and a specificity of 87.9%. With a cutoff of 14.4% for post-menopausal patients, the test had a sensitivity of 90.8% and a specificity of 66.3%. For EOC *vs* benign disease, the ROC–AUC of ROMA increased to 0.913 and for invasive EOC *vs* benign disease to 0.957.

**Conclusion:**

This independent validation study demonstrated similar performance indices to those recently published. However, in this study, HE4 and ROMA did not increase the detection of malignant disease compared with CA125 alone. Although the initial reports were promising, measurement of HE4 serum levels does not contribute to the diagnosis of ovarian cancer.

The majority of women who undergo surgery for an ovarian cyst or pelvic mass are treated in a community hospital by a gynaecologist or general surgeon. Although this is appropriate for patients who have a benign cyst, patients with a malignancy should be referred to a tertiary care centre with multidisciplinary teams specialised in ovarian cancer treatment. A recent systematic review showed an improved outcome for patients with ovarian cancer when they were referred to, and surgically treated by, gynaecological oncologists (du Bois *et al*, 2009). Therefore, it is important to triage women with increased risk for ovarian cancer to the appropriate surgeon and centre.

CA125 is the most widely used tumour marker in ovarian cancer (Bast *et al*, 1983). The sensitivity and specificity of CA125 are far from ideal as its levels are raised in approximately 80% of all epithelial ovarian cancers (EOC) and in only 50% of stage I EOC (Zurawski *et al*, 1988). Therefore, CA125 is rarely used as a unique parameter in the prediction of malignancy. Usually, a combination of a patient's medical history, clinical examination results, imaging data and tumour marker profile is used to differentiate malignant ovarian masses from their benign counterparts. Ultrasound has an important role in differentiating between benign and malignant adnexal masses, but experience and proper training are of paramount importance in distinguishing both adnexal masses (Van Holsbeke *et al*, 2009). This highlights a major problem in that the centre with the least experience in dealing with malignant disease requires substantial experience in ultrasound to triage patients to a gynaecological oncologist. This explains the tremendous amount of effort that has been expended over the past few decades to find new ovarian cancer biomarkers that could be used together with, or instead of, CA125. In 1999, the *human epididymis secretory protein 4* (*HE4*) gene was found to be overexpressed in ovarian cancer (Schummer *et al*, 1999). It is a member of the *Wey acidic protein* gene family (Bouchard *et al*, 2006), and is expressed in normal tissues of the reproductive and respiratory tract (Bingle *et al*, 2002; Galgano *et al*, 2006). The first report mentioning HE4 as a potential serum biomarker for ovarian cancer was published in 2003 (Hellstrom *et al*, 2003). Recently, Moore *et al* (2008b, 2009) published a series of papers that used a combination of CA125, HE4 and menopausal status to predict the presence of a malignant ovarian tumour. Originally, nine potential biomarkers were evaluated, of which HE4 was the most effective in detecting ovarian cancer. When CA125 was combined with HE4, the prediction rate was higher, showing a sensitivity for detecting malignant disease of 76.4% at a specificity of 95% (Moore *et al*, 2008b). Subsequently, Moore *et al* (2009) performed a multicentre prospective study including 531 women diagnosed with a pelvic mass who underwent surgery. Patients were classified as being at a high or low risk for ovarian cancer with a specificity of 75.0% and a sensitivity of 92.3% for post-menopausal patients, and a specificity and sensitivity of 74.8 and 76.5%, respectively, for pre-menopausal patients.

In this study, we aimed to independently validate HE4 and the combination of HE4 with CA125 using the Risk of Ovarian Malignancy Algorithm (ROMA) for the diagnosis of ovarian cancer.

## PATIENTS AND METHODS

### Patients

From August 2005 to March 2009, 389 patients were included in a prospective study conducted at the University Hospitals Leuven. All patients were diagnosed with a pelvic mass of suspected ovarian origin and were scheduled for surgical intervention. Women with a previous bilateral oophorectomy were not eligible. All patients underwent imaging by pelvic ultrasound to document the presence of an ovarian mass. Clinical information was retrieved from the patients’ hospital notes. All patients underwent surgical removal of the ovarian mass, and if a patient was diagnosed with an ovarian cancer, then surgical staging was performed.

Before the collection of biological samples and surgery, all patients were required to give fully informed consent. The protocol was approved by the Local Ethics Committee. The Ethical Committee released the authors from the obligation to obtain an insurance contract because of the character of this study. Patient participation in the study was concluded once the final surgical pathology reports were obtained.

### Serum samples

Immediately before surgery, blood samples were obtained. Blood samples were collected in 10 ml clothing activating tubes (BD Vacutainer Serum Tube, ref. 369033; Becton-Dickinson, Erembodegem, Belgium). Serum tubes were centrifuged at 800 **g** for 10 min. Serum was collected, dispensed into multiple cryotubes and frozen at −80 °C. The time between blood sampling and freezing of the serum and presence of haemolysis was noted. The targeted time limit between sampling and freezing was 4 h.

### Marker assays

Serum CA125 concentrations were measured using the CanAg CA125 EIA assay (Fujirebio Diagnostics, Göteborg, Sweden) and serum HE4 concentrations were measured using the HE4 EIA assay (Fujirebio Diagnostics). Both assays are solid-phase, non-competitive immunoassays, based on the direct sandwich technique, and were run according to manufacturer's instructions. Each ELISA was performed manually and in duplicate for calibrators, controls and patient samples. The appropriate controls were within the ranges provided by the manufacturer for all runs. For CA125, the normal upper limit was 35 U ml^−1^, whereas that for HE4 was 70 pM (as suggested by Moore *et al* (2008b) or 150 pM (as suggested in the product insert). A cutoff point that provided the best accuracy (minimal false-negative and false-positive results) in the study was also determined. We also determined our own ideal cutoff, corresponding to the highest accuracy.

### Statistical analysis

ROMA classifies patients as being at a low or at a high risk for malignant disease using the following algorithms:

- Premenopausal: predictive index (PI)=−12.0+(2.38 × LN(HE4))+(0.0626 × LN(CA125))
- Postmenopausal: PI=−8.09+(1.04 × LN(HE4))+[0.732 × LN(CA125))
- Predicted probability: (PP)=100 × exp(PI)/(1+exp(PI))

According to the manufacturer's insert, the following thresholds were selected for ROMA:

- Pre-menopausal women:
  - PP ⩾12.5%=high risk of finding EOC
  - PP <12.5%=low risk of finding EOC
  - Post-menopausal women:
    - PP ⩾14.4%=high risk of finding EOC
    - PP <14.4%=low risk of finding EOC

Statistical analysis was performed with MedCalc v11.1.1.0 (MedCalc Software, Mariakerke, Belgium) and with PASW Statistics v17.0 (SPSS, Brussels, Belgium). The mean age of the patients was compared using Student's *t*-test, and categorical variables were compared with the *χ*^2^-test. Tumour marker levels were compared using the Wilcoxon rank-sum test (Mann–Whitney two sample statistic) or the Kruskal–Wallis rank test (multiple sample statistic).

Receiver operator characteristic (ROC) curves were constructed, and the area under the curve (ROC–AUC) with a 95% confidence interval was calculated. Sensitivity and specificity were calculated in pre- and post-menopausal women separately and independently of menopausal status. Subgroups were analysed according to the histological subtype of the tumour, stage, grade, use of hormonal drugs, smoking habit, familial history, presence of haemolysis and the time between sampling and freezing. The method described by DeLong *et al* (1988) was used for the calculation of the difference between two ROC–AUCs. For all statistical comparisons, a *P*-value of <0.05 was considered statistically significant.

## RESULTS

### Patient characteristics

The serum of 389 patients was analysed: 228 (58.6%) patients had benign disease and 161 (41.4%) patients had malignant disease (Table 1). Patients with benign disease were younger and more likely to be pre-menopausal. Patients with malignant disease were more likely to have a family history of breast and ovarian cancer.

### Sample characteristics

Overall, 40 samples were not frozen within a time limit of 4 h after sampling, of which 24 (10.5%) were from benign cases and 16 (9.9%) were from malignant cases (*P*=0.851). Haemolysis was noted in 38 cases, of which 23 (10.1%) were from benign cases and 15 (9.3%) were from malignant cases (*P*=0.801).

### Tumour characteristics

The most common benign ovarian tumours were cystadenomas (*n*=52), cystadenofibromas (*n*=26), endometriomas (*n*=66) and mature teratomas (*n*=29) (Tables 2 and 3). Mixed tumours (*n*=16) contain two or more different histological subtypes, making it impossible to categorise these tumours into a specific subtype. The cystadenomas and cystadenofibromas included 47 serous, 26 mucinous and 5 other histological types or mixed cystadenomas/cystadenofibromas. The majority of the malignant tumours were EOC. Most of the EOC were of high grade and were diagnosed at an advanced stage. Other primary non-epithelial ovarian tumours (NEOCs) included two sex cord stromal tumours and two sarcomas. All other malignant tumours of the ovary (*n*=26) were metastases from extra-ovarian primary tumours. These tumours were mainly of an endometrial or gastrointestinal origin.

### Tumour marker levels

**Figure 1:**
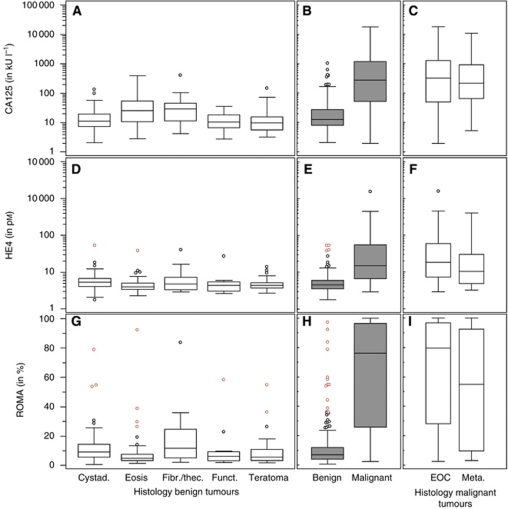
Box and whisker plots representing median levels and the interquartile range (box) of CA125 (in U ml^−1^), HE4 (in pM) and ROMA (in %) for benign histology subtypes (subset (**A**), (**D**) and (**G**)), benign *vs* malignant (subset (**B**), (**E**) and (**H**)) and malignant histology subtypes (subset (**C**), (**F**) and (**I**)). The *y* axis is a logarithmic scale for CA125 and HE4. Abbreviations: Cystad.=cystadenoma or cystadeanofibroma; Eosis=endometriosis; Fibr./thec.=ovarian fibroma or thecoma; Funct.=functional cyst; EOC=epithelial ovarian cancer; Meta.=metastatic disease to the ovary.

**Figure 2:**
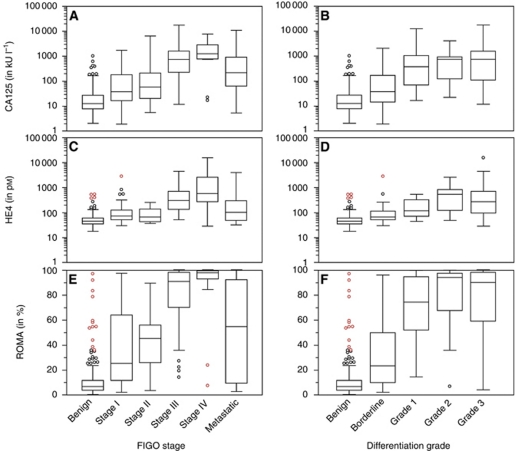
Box and whisker plots representing median levels and the interquartile range (box) of CA125 (in U ml^−1^), HE4 (in pM) and ROMA (in %) at various FIGO stages (subset (**A**), (**C**) and (**E**)) and various grades, including borderline (subset (**B**), (**D**) and (**F**)). The *y* axis is a logarithmic scale for CA125 and HE4.

The median CA125, HE4 and ROMA serum levels differed significantly between benign and malignant cases for the whole group, and for the pre- and post-menopausal groups separately (*P*<0.0001 for all comparisons) (Table 4, Figures 1 and 2). Within the benign group, the most frequent tumours were analysed. Using Kruskal–Wallis rank test, we found the median tumour marker levels to be statistically different for CA125 (*P*<0.0001), HE4 (*P*=0.0043) and ROMA (*P*=0.0006). *Post hoc* pairwise comparisons with the Wilcoxon rank-sum test showed that CA125 was significantly elevated in endometriosis and ovarian fibromas/thecomas compared with cystadenomas/cystadenofibromas (*P*<0.0001 and *P*=0.0111), functional cysts (*P*=0.0160 and *P*=0.0281) and mature teratomas (*P*=0.0002 and *P*=0.0169). For HE4, the only significant comparison found was the pairwise comparison between cystadenomas/cystadenofibromas and endometriosis (*P*=0.0002). Risk of Ovarian Malignancy Algorithm was significantly elevated in cystadenomas/cystadenofibromas (*P*<0.0001) and ovarian fibromas/thecomas (*P*=0.0111) when compared with endometriosis, and in cystadenomas/cystadenofibromas when compared with mature teratomas (*P*=0.0349).

Within the group of malignant tumours, there was no significant difference between the CA125, HE4 and ROMA levels of EOC and metastatic tumours. There was no significant difference between FIGO stages I and II tumours, nor between FIGO stages III and IV tumours, although the difference between early (FIGO I–II) and advanced stages (FIGO III–IV) was significant for CA125, HE4 and ROMA. There was a significant difference between borderline and invasive disease (grades 1–3) for all markers, but there was no difference among grades 1, 2 and 3 for the different markers.

### ROC curves and performance indices for all tumours

**Figure 3:**
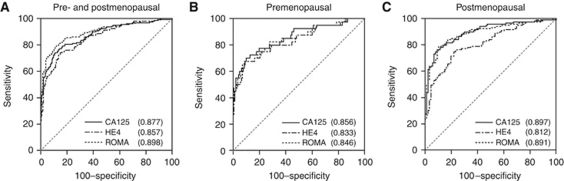
ROC curves for CA125, HE4 and ROMA among patients with all types and stages of ovarian tumours. (**A**) Malignant *vs* benign disease in pre- and post-menopausal patients together. (**B**) Malignant *vs* benign disease in pre-menopausal patients. (**C**) Malignant *vs* benign disease in post-menopausal patients. Total AUC values for each assay are listed in parentheses.

The ROC–AUC of CA125 was not significantly different from that of HE4 or ROMA for all malignant diseases (including EOC, NEOC and metastases) compared with benign disease (Table 5, Figure 3). Pairwise comparison of ROC–AUCs showed that only the difference between HE4 and ROMA was significant. For pre-menopausal patients, again, only the pairwise comparison between HE4 and ROMA was significant. In the post-menopausal population, there was a significantly better performance of CA125 *vs* HE4, and of ROMA *vs* HE4. Overall, ROMA did not perform significantly better than CA125 alone, either for the whole group of patients or for the pre- or post-menopausal patients separately.

At the ideal cutoff, corresponding to the highest accuracy (minimal false-negative and false-positive results), CA125, HE4 and ROMA resulted in a similar sensitivity and specificity within the different menopausal groups. Sensitivity and specificity using the cutoff values in the manufacturer's protocol for HE4 and ROMA are also shown in Table 5, together with the sensitivity and specificity of HE4 at a cutoff of 70 pM.

### ROC curves for subgroups

**Figure 4:**
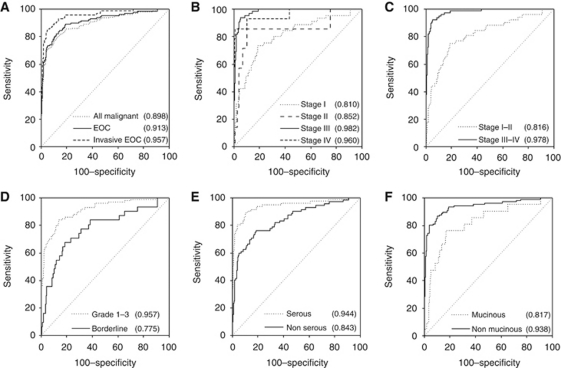
ROC curves for ROMA. The ROC curves in the different subsets represent different groups of ovarian cancer (cases) compared with benign ovarian tumours (non-cases). (**A**) All malignant tumours, epithelial ovarian cancer (EOC) and invasive EOC (excluding borderline tumours) *vs* benign disease. (**B**) FIGO stages I, II, III and IV *vs* benign disease. (**C**) Early stage (stages I and II combined) and advanced stage (stages III and IV combined) *vs* benign disease. (**D**) Grades 1–3 EOC and borderline EOC *vs* benign disease. (**E**) Serous EOC and non-serous EOC *vs* benign disease (all mixed serous tumours were excluded from this analysis). (**F**) Mucinous EOC and non-mucinous EOC *vs* benign disease (all mixed mucinous tumours were excluded from this analysis). Total AUC values for each assay are listed in parentheses.

When EOC was analysed alone and NEOC and metastatic tumours were excluded, the ROC–AUC of ROMA was higher (Figure 4, Supplementary Table 1). The ROC–AUC was even higher when all borderline tumours were excluded. With regard to histological subtypes, a comparison of ROC–AUC of (pure) serous with that of non-serous EOC (excluding all mixed serous tumours) showed that ROC–AUC of ROMA was higher for the serous subtype. In contrast, all markers performed significantly worse when only the mucinous subtype was examined.

## DISCUSSION

This study aimed to investigate the performance of serum tumour markers CA125 and HE4, and the risk stratification tool ROMA in a prospective collection of serum samples from patients with an ovarian mass. We found that there was a significant difference between benign and malignant disease with respect to serum CA125, HE4 and ROMA levels. When the ROC–AUCs of the different tumour markers were compared, HE4 and CA125 performed similarly, except for the post-menopausal patients in whom CA125 performed better. This similar performance of HE4 and CA125 was also noted in other studies (Hellstrom *et al*, 2003; Scholler *et al*, 2006; Palmer *et al*, 2008; Montagnana *et al*, 2009; Andersen *et al*, 2010). Combining HE4 and CA125 in the ROMA improved HE4 but not CA125 performance, regardless of menopausal status. As CA125 is the current standard for comparison, this means that neither HE4 nor the ROMA improved the diagnosis of ovarian cancer. This is in contrast to the results of Moore *et al* (2008b), who found that a combination of CA125 and HE4 performed better than CA125 alone. However, Moore *et al* (2008b) excluded all borderline tumours, NEOC and metastatic tumours to calculate the performance of the tumour markers they tested. We decided not to exclude these tumours in our initial analysis as we wanted to study a patient population that reflected a normal clinical setting. When borderline tumours, NEOC and metastatic cancers were excluded, the ROC–AUC for CA125 was 0.937 *vs* 0.836 in the study by Moore *et al* (2008b). In contrast, the ROC–AUCs of HE4 were similar: 0.914 (our data) *vs* 0.908 (Moore *et al*, 2008b). In a more recent study, Moore *et al* (2010) also included borderline tumours in their analysis. Within this study, the examination of benign cases *vs* all stages of EOC and borderline tumours revealed an ROC–AUC of 0.913. Within a setting of a multicentre prospective trial with central review and monitoring it seems plausible that a diagnostic test would perform slightly better.

Compared with CA125, HE4 is inversely influenced by age; whereas CA125 is higher in healthy pre-menopausal patients (Bon *et al*, 1996; Bonfrer *et al*, 1997), HE4 tends to be higher in post-menopausal patients (Moore *et al*, 2008a, 2008b; Andersen *et al*, 2010). These slightly higher normal values influence the performance of the tumour markers concerned. Although not significant, this can also be seen in our study population: the ROC–AUC of CA125 was higher in the post-menopausal group. Of particular interest, HE4 seems to have a slightly higher ROC–AUC in the pre-menopausal group than in the post-menopausal group. Although this difference is not significant, it causes the ROC curves of CA125 and HE4 to come together in the pre-menopausal group and diverge in the post-menopausal group (Figure 3). In other words, the performance of HE4 is similar to that of CA125 in the pre-menopausal group, but significantly worse in the post-menopausal group. This increased performance of HE4 in the pre-menopausal group is in agreement with previous studies (Moore *et al*, 2008b; Andersen *et al*, 2010), and confirms that CA125 and HE4 function independently of each other.

Owing to the fact that ROC curves are not used in clinical practice, we aimed to find the cutoff points for the different tumour markers. The cutoff values corresponding to the highest accuracy (minimal false-negative and false-positive results) for all patients were 62.5 kU l^−1^ for CA125, 72.2 pM for HE4 and 22.2% for ROMA. In the product insert, it is suggested that 94.4% of the healthy female subjects (*n*=179) that were studied had a HE4 value of 150 pM or below. If we define the reference value as the value that includes 95% of healthy controls, and we use this as a cutoff point to minimise the false-positive rate, we obtain a sensitivity of 50.3% and a specificity of 96.5%. In clinical practice, this means that 3.5% of patients with a benign tumour will be treated as if they had a malignant tumour (overtreatment), and 49.7% of patients with a malignant tumour will be treated as if they had a benign tumour (undertreatment). Therefore, in our study, this cutoff point is not useful for differentiating benign from malignant cysts. Andersen *et al* (2010) also determined their cutoff at the 95th percentile in a healthy control group. On the basis of this cutoff, they obtained a sensitivity of 77.0% and a specificity of 94.9%. Unfortunately, they failed to mention what their cutoff value was. Using the cutoff point of 70 pM, as previously suggested by Moore *et al* (2008b), we reached a sensitivity of 74.5% and a specificity of 83.3%. This is therefore comparable to our ideal cutoff point of 72.2 pM, and is thus a reasonable cutoff point for HE4. With regard to ROMA, different cutoff points are used in pre-menopausal and post-menopausal patients. Both cutoff points are determined to provide a specificity level of 75% for the CA125 plus HE4 assay combination. Our ideal cutoff points of 16.6% for the pre-menopausal patients and 35.9% for the post-menopausal patients were somewhat different from those suggested previously. However, these were not established at 75% specificity, but at the point on the ROC curve at which we had minimal false-negative and false-positive results. Irrespective of whether we analyse only invasive EOC, our ideal cutoff point in the pre-menopausal and post-menopausal category is higher than the suggested cutoff points of 12.5 and 14.4%, respectively.

As expected, histological subtypes seem to be important for the performance of the different tumour markers. With regard to benign tumours, it was interesting to see that the fibromas/thecomas group and the endometriomas had the highest levels of CA125, whereas for HE4, the endometriomas had the lowest level. As already mentioned by Huhtinen *et al* (2009), measuring both CA125 and HE4 together could be of particular interest in differentiating endometriosis from ovarian cancer, as ovarian cancer will cause a raised CA125 and HE4, whereas endometriosis will only cause a raised CA125. This could explain why HE4 performs better in pre-menopausal patients compared with post-menopausal patients, and *vice versa* for CA125. However, even for the pre-menopausal patients, HE4 and ROMA did not perform better than CA125. All malignant tumours expressed high levels of CA125 and HE4, but the highest levels were noted for the serous subtype. High expression levels of HE4 for the different epithelial subtypes, with the exception of the mucinous subtype, were already noticed in previous studies (Lu *et al*, 2004; Drapkin *et al*, 2005; Gilks *et al*, 2005; Galgano *et al*, 2006).

Although CA125, HE4 and ROMA are not currently recommended as a screening tool, it is interesting to see how well a tumour marker performs in the early stage of disease. A definite trend could be seen from stage I to stage IV disease, and CA125 and HE4 performed significantly worse when early and late stages of disease were compared. As a consequence, the ROMA also performed worse. With these ROC–AUCs, the chances that HE4 or ROMA will be successful as a screening marker are low, as very high specificities are required in screening for low prevalent disease.

In summary, this large independent validation study was able to demonstrate similar performance indices as those recently published in the literature. However, in our study, neither HE4 nor ROMA increased the detection of malignant disease. Human Epididymis secretory protein 4, or its combination with CA125, could be useful in diagnosing certain benign or malignant subtypes; however, this needs to be explored in more detail.

## Figures and Tables

**Table 1:** Distribution of patient characteristics for patients with a benign or malignant pelvic mass

**Variable**	**Numerical display**	**Benign**	**Malignant**	***P*-value**
Number of cases	*n* (%)	228 (58.6)	161 (41.4)	NA
Age (in years)	Mean (s.d.)	46.3 (16.0)	57.8 (12.6)	<0.001
Post-menopausal	*n* (%)	86 (37.7)	119 (73.9)	<0.001
Smoking	*n* (%)	53 (23.2)	31 (19.3)	0.457
OC^a^	*n* (%)	44 (31.2)	12 (30.0)	0.682
HRT^b^	*n* (%)	14 (16.3)	9 (7.6)	0.053
				
*Family history*				
Breast cancer	*n* (%)	35 (15.4)	41 (25.5)	0.009
Ovarian cancer	*n* (%)	4 (1.8)	8 (5.0)	0.028

Abbreviations: HRT=hormone replacement therapy; OC=oral contraception; NA=not applicable.

a For pre-menopausal patients.

b For post-menopausal patients.

**Table 2:** Histological type and distribution of benign disease

**Histological type**	***n***	**%**
Cystadenoma/cystadenofibroma^a^	78	34.1
Endometriosis	66	28.9
Mature teratoma	29	12.7
Fibroma/thecoma	15	6.6
Functional cyst	13	5.7
Hydrosalpinx	3	1.3
Abces	2	0.9
Parasalpingeal cyst	2	0.9
Struma ovarii	2	0.9
Leydig cell tumour	1	0.4
Unknown	1	0.4
Mixed	16	7.0
Total	228	100.0

a Including 47 serous, 26 mucinous and 5 other or mixed cystadenomas/cystadenofibromas.

**Table 3:** FIGO stage, differentiation grade and histological type of malignant disease

	***n***	**%**
*Histological type*		
Epithelial	131	81.4
Serous	84	52.2
Mucinous	21	13.0
Endometrioid	7	4.3
Clear cell	6	3.7
Mixed	6	3.7
Carcinosarcoma	4	2.5
Undifferentiated	3	1.9
Granulosa cell	2	1.2
Sarcoma	2	1.2
Metastatic	26	16.1
Endometrium	11	6.8
Colon	5	3.1
Appendix	3	1.9
Mesothelioma	2	1.2
Breast	1	0.6
Lung	1	0.6
Lymphoma	1	0.6
Pancreas	1	0.6
Stomach	1	0.6
*Total*	161	100.0
		
*FIGO stage* a		
I	43	32.8
II	8	6.1
III	66	50.4
IV	14	10.7
Total	131	100.0
		
*Differentiation grade* a		
Borderline	31	23.7
Grade 1	13	9.9
Grade 2	14	10.7
Grade 3	73	55.8
Total	131	100.*0*

Abbreviation: FIGO=International Federation of Gynecology and Obsterics.

a For epithelial ovarian cancer only.

**Table 4:** Serum CA125, HE4 and ROMA levels according to histology, FIGO stage and tumour grade

	**CA125 (U ml^−1^)**		**HE4 (pM)**		**ROMA (%)**	
	**Median**	**IR**	**Median**	**IR**	**Median**	**IR**
*Benign histology*	12.8	8.0–27.6	45.4	35.6–60.8	6.8	3.9–11.9
Cystadenoma/cystadenofibroma	11.3	7.4–19.5	53.7	40.8–68.0	9.1	5.7–14.4
Endometriosis	25.5	10.7–54.9	40.0	34.3–50.7	4.9	3.3–7.5
Fibroma/thecoma	29.1	11.6–45.5	48.1	33.9–73.2	11.6	5.0–24.7
Functional cyst	10.5	6.8–20.7	43.7	30.8–54.6	6.2	3.0–9.4
Mature teratoma	9.8	5.7–15.9	43.9	37.5–52.5	5.6	3.4–10.9
						
*Malignant histology*	276.5	52.4–1195.9	151.8	67.4–560.4	76.1	25.7–96.5
Epithelial ovarian cancer	321.3	50.2–1291.4	184.6	72.9–589.3	79.6	28.1–96.7
Metastatic	222.9	64.9–913.5	103.5	48.9–302.4	55.0	9.7–92.5
*FIGO stage*						
I	38.4	16.9–182.6	73.2	52.6–126.5	25.6	11.8–64.3
II	60.2	20.6–254.8	69.0	44.3–152.6	45.5	26.1–56.2
III	757.3	227.9–1640.0	308.0	135.0–712.5	91.2	70.3–98.4
IV	1260.7	790.6–2905.1	578.7	274.6–2612.9	98.0	93.0–99.5
*Tumour grade*						
Borderline	38.3	14.8–170.4	67.6	52.6–116.4	23.4	10.0–50.1
Grade 1	379.4	83.2–1120.4	119.9	72.7–336.5	74.5	52.2–94.9
Grade 2	755.7	124.2–945.4	552.9	125.0–844.3	94.3	67.8–97.6
Grade 3	748.7	112.1–1580.9	274.6	97.8–716.7	90.3	59.2–98.4

Abbreviations: FIGO=International Federation of Gynecology and Obstetrics; HE4=human epididymis secretory protein 4; IR=interquartile range; ROMA=Risk of Ovarian Malignancy Algorithm.

**Table 5:** Comparison of ROC–AUCs, sensitivity and specificity for CA125 (U ml^−1^), HE4 (pM) and ROMA (%) among patients with all types and stages of ovarian tumours

			**Pairwise comparison of ROC–AUC^a^**			**Ideal cutoff^b^**			**Suggested cutoff**		
**Menopausal status**	**Marker**	**ROC–AUC (95% CI)**	**HE4 *vs* CA125**	**HE4 *vs* ROMA**	**CA125 *vs* ROMA**	**Cutoff**	**Sensitivity (%)**	**Specificity (%)**	**Cutoff**	**Sensitivity (%)**	**Specificity (%)**
All patients	ROMA	0.898 (0.863–0.926)	*P*=0.306	*P*<0.001	*P*=0.172	22.2	79.2	88.1	12.5/14.4^c^	84.9	79.7
	CA125	0.877 (0.840–0.908)				62.5	73.9	89.0	35.0	79.5	81.6
	HE4	0.857 0.819–0.891)				72.2	73.9	85.1	70.0^d^	74.5	83.3
									150.0^e^	50.3	96.5
Pre-menopausal	ROMA	0.846 (0.785–0.895)	*P*=0.570	*P*=0.044	*P*=0.782	16.6	67.5	91.5	12.5^c^	67.5	87.9
	CA125	0.856 (0.796–0.904)				83.8	70.0	90.1	35.0	75.0	80.1
	HE4	0.833 (0.771–0.885)				66.0	67.5	90.8	70.0^d^	67.5	90.8
									150.0^e^	42.5	99.3
Post-menopausal	ROMA	0.891 (0.840–0.930)	*P*=0.001	*P*<0.001	*P*=0.487	35.9	79.0	89.5	14.4^c^	90.8	66.3
	CA125	0.897 (0.847–0.935)				51.2	76.5	90.7	35.0	81.5	83.7
	HE4	0.812 (0.752–0.863)				74.2	74.8	77.9	70.0^d^	77.3	70.9
									150.0^e^	52.9	91.9

Abbreviations: CI=confidence interval; HE4=human epididymis secretory protein 4; ROC–AUC=receiver operator characteristic–area under the curve; ROMA=Risk of Ovarian Malignancy Algorithm.

a Differences in ROC–AUCs were calculated by using the method as described by DeLong *et al*, 1988.

b Cutoff value corresponding to the highest accuracy (minimal false-negative and false-positive results).

c Cutoff values for ROMA: 12.5% for the pre-menopausal patients and 14.4% for the post-menopausal patients, as suggested in the product insert.

d Cutoff value for HE4 at 70 pM as suggested by Moore *et al*, (2008b).

e Cutoff value for HE4 at 150 pM as suggested in the product insert (17).
