# Supplementary material for: HE4 and CA125 as a diagnostic test in ovarian cancer: prospective validation of the Risk of Ovarian Malignancy Algorithm
Source: Br J Cancer. 2011 Feb 8;104(5):863–70. doi: 10.1038/sj.bjc.6606092 (PMC3048204; doi:10.1038/sj.bjc.6606092)
Supplement: Supplementary Information [file 6606092x1.doc]

| Supplementary Table 1: Comparison of ROC-AUCs for CA125, HE4 and ROMA among different patient subgroups according to tumour, personal and sample characteristics. | | | | | | | | | | | | |
| --- | --- | --- | --- | --- | --- | --- | --- | --- | --- | --- | --- | --- |
|  | |  | | HE4 |  |  | CA125 |  |  | ROMA |  |  |
|  | |  | | AUC | 95%CI | p | AUC | 95%CI | p | AUC | 95%CI | p |
| Type of cancer | |  | |  |  |  |  |  |  |  |  |  |
|  | All malignant^*^ | | 0.857 | | 0.818-0.890 | NA | 0.877 | 0.840-0.908 | NA | 0.898 | 0.863-0.926 | NA |
|  | EOC^†^ | | 0.871 | | 0.831-0.903 |  | 0.886 | 0.848-0.917 |  | 0.913 | 0.879-0.941 |  |
|  | Invasive EOC^‡^ | | 0.914 | | 0.878-0.942 |  | 0.937 | 0.905-0.961 |  | 0.957 | 0.929-0.976 |  |
|  | |  | |  |  |  |  |  |  |  |  |  |
| FIGO Stage | |  | |  |  |  |  |  |  |  |  |  |
|  | I | | 0.767 | | 0.713-0.816 | NA | 0.755 | 0.700-0.805 | NA | 0.810 | 0.758-0.855 | NA |
|  | II | | 0.705 | | 0.642-0.762 |  | 0.769 | 0.710-0.821 |  | 0.852 | 0.800-0.895 |  |
|  | III | | 0.954 | | 0.923-0.975 |  | 0.969 | 0.943-0.986 |  | 0.982 | 0.959-0.994 |  |
|  | IV | | 0.914 | | 0.871-0.946 |  | 0.950 | 0.915-0.974 |  | 0.960 | 0.927-0.981 |  |
|  | |  | |  |  |  |  |  |  |  |  |  |
| Stage | |  | |  |  |  |  |  |  |  |  |  |
|  | Early (I-II) | | 0.758 | | 0.704-0.807 | <0.0001 | 0.757 | 0.703-0.806 | <0.0001 | 0.816 | 0.765-0.859 | <0.0001 |
|  | Late (III-IV) | | 0.947 | | 0.916-0.969 |  | 0.966 | 0.939-0.983 |  | 0.978 | 0.955-0.991 |  |
|  | |  | |  |  |  |  |  |  |  |  |  |
| Histology | |  | |  |  |  |  |  |  |  |  |  |
|  | Serous | | 0.910 | | 0.873-0.939 | NA | 0.910 | 0.873-0.939 | NA | 0.944 | 0.912-0.967 | NA |
|  | Endometrioid | | 0.826 | | 0.771-0.872 |  | 0.878 | 0.830-0.917 |  | 0.939 | 0.900-0.966 |  |
|  | Clear cell | | 0.824 | | 0.769-0.870 |  | 0.900 | 0.854-0.935 |  | 0.846 | 0.793-0.890 |  |
|  | Mucinous | | 0.748 | | 0.689-0.800 |  | 0.794 | 0.738-0.842 |  | 0.817 | 0.763-0.863 |  |
| Histology | |  | |  |  |  |  |  |  |  |  |  |
|  | Serous | | 0.910 | | 0.873-0.939 | 0.0324 | 0.910 | 0.873-0.939 | 0.0855 | 0.944 | 0.912-0.967 | 0.0028 |
|  | Non-serous | | 0.777 | | 0.725-0.823 |  | 0.840 | 0.794-0.880 |  | 0.843 | 0.797-0.882 |  |
| Histology | |  | |  |  |  |  |  |  |  |  |  |
|  | Mucinous | | 0.748 | | 0.689-0.800 | 0.0072 | 0.794 | 0.738-0.842 | 0.0123 | 0.817 | 0.763-0.863 | 0.0299 |
|  | Non-mucinous | | 0.903 | | 0.866-0.933 |  | 0.911 | 0.875-0.939 |  | 0.938 | 0.906-0.961 |  |
| Tumour grade | |  | |  |  |  |  |  |  |  |  |  |
|  | Grade 1 | | 0.883 | | 0.836-0.921 | NA | 0.903 | 0.858-0.937 | NA | 0.954 | 0.920-0.977 | NA |
|  | Grade 2 | | 0.929 | | 0.889-0.958 |  | 0.956 | 0.921-0.978 |  | 0.957 | 0.923-0.979 |  |
|  | Grade 3 | | 0.914 | | 0.876-0.943 |  | 0.938 | 0.904-0.962 |  | 0.958 | 0.928-0.978 |  |
| Tumour grade | |  | |  |  |  |  |  |  |  |  |  |
|  | Grade 1-3 | | 0.912 | | 0.876-0.940 | 0.0004 | 0.936 | 0.903-0.960 | 0.0001 | 0.957 | 0.929-0.976 | 0.0004 |
|  | Borderline | | 0.737 | | 0.679-0.790 |  | 0.726 | 0.667-0.779 |  | 0.775 | 0.719-0.825 |  |
| Family history | |  | |  |  |  |  |  |  |  |  |  |
|  | Yes | | 0.832 | | 0.733-0.905 | 0.4938 | 0.878 | 0.788-0.940 | 0.8951 | 0.853 | 0.757-0.922 | 0.2202 |
|  | No | | 0.867 | | 0.823-0.904 |  | 0.872 | 0.827-0.908 |  | 0.913 | 0.875-0.943 |  |
| Smoking | |  | |  |  |  |  |  |  |  |  |  |
|  | Yes | | 0.825 | | 0.727-0.899 | 0.3629 | 0.871 | 0.780-0.934 | 0.8144 | 0.890 | 0.802-0.948 | 0.6866 |
|  | No | | 0.873 | | 0.829-0.908 |  | 0.882 | 0.840-0.917 |  | 0.906 | 0.867-0.937 |  |
| OC | |  | |  |  |  |  |  |  |  |  |  |
|  | Yes | | 0.820 | | 0.694-0.910 | 0.6328 | 0.843 | 0.721-0.926 | 0.9149 | 0.839 | 0.716-0.924 | 0.7291 |
|  | No | | 0.868 | | 0.785-0.927 |  | 0.853 | 0.768-0.916 |  | 0.873 | 0.792-0.931 |  |
| HRT | |  | |  |  |  |  |  |  |  |  |  |
|  | Yes | | 0.683 | | 0.458-0.859 | 0.2102 | 0.810 | 0.593-0.941 | 0.3618 | 0.778 | 0.558-0.922 | 0.2681 |
|  | No | | 0.844 | | 0.779-0.896 |  | 0.908 | 0.852-0.947 |  | 0.910 | 0.854-0.949 |  |
| Haemolysis | |  | |  |  |  |  |  |  |  |  |  |
|  | Yes | | 0.800 | | 0.663-0.900 | 0.3416 | 0.907 | 0.790-0.970 | 0.4716 | 0.853 | 0.725-0.937 | 0.4031 |
|  | No | | 0.864 | | 0.823-0.899 |  | 0.874 | 0.834-0.908 |  | 0.903 | 0.866-0.933 |  |
| Freezing within 1-4h | |  | |  |  |  |  |  |  |  |  |  |
|  | Yes | | 0.860 | | 0.819-0.894 | 0.9053 | 0.878 | 0.839-0.910 | 0.9625 | 0.896 | 0.859-0.926 | 0.8247 |
|  | No | | 0.852 | | 0.700-0.946 |  | 0.875 | 0.727-0.960 |  | 0.908 | 0.766-0.978 |  |
|  | |  | |  |  |  |  |  |  |  |  |  |
| Abbreviations: EOC: epithelial ovarian cancer; OC: oral contraceptives; HRT: hormone replacement therapy  Differences in ROC-AUCs were calculated according to DeLong et al. (18)  ^*^ Including EOC, non-epithelial ovarian cancers and metastatic cancers to the ovary.  ^†^ Including borderline or invasive EOC  ^‡^ Including only invasive EOC | | | | | | | | | | | | |
